# Supplementary material for: 4D printed deformation labels with machine learning for monitoring and preservation of respiring climacteric fruits
Source: Nat Commun. 2025 Nov 21;16:11525. doi: 10.1038/s41467-025-66554-6 (PMC12749378; doi:10.1038/s41467-025-66554-6)
Supplement: Supplementary file 1 — Supplementary Information [file 41467_2025_66554_MOESM1_ESM.pdf]

Supplementary Information

4D printed deformation labels with machine learning for monitoring and preservation of respiring climacteric fruits

Xiuxiu Teng [1,3], Min Zhang [1,2*], Arun S. Mujumdar [4], Chunli Li [1]

[1] State Key Laboratory of Food Science and Resources, School of Food Science and Technology, Jiangnan University, 214122 Wuxi, Jiangsu, China

[2] Jiangsu Province International Joint Laboratory on Fresh Food Smart Processing and Quality Monitoring, Jiangnan University, 214122 Wuxi, Jiangsu, China

[3] China General Chamber of Commerce Key Laboratory on Fresh Food Processing & Preservation, Jiangnan University, 214122 Wuxi, Jiangsu, China

[4] Department of Bioresource Engineering, Macdonald Campus, McGill University, Quebec, Canada

*Corresponding author: Professor Min Zhang, School of Food Science and Technology, Jiangnan University, 214122 Wuxi, Jiangsu Province, China.

Tel.: 0086-510-85877225; Fax: 0086-(0)510-85807976

E-mail: min@jiangnan.edu.cn

Supplementary Table 1 The major chemical compositions of blueberry anthocyanins and un-extractable hawthorn polyphenols. All data are mean ± S.D. n = 3 independent samples per group.

Sample	Compositions	Content (mg/g)
Blueberry anthocyanins	Delphinidin	54.73 ± 9.13
	Cyanidin	165.45 ± 10.86
	Petunidin	190.82 ± 8.90
	Peonidin	243.75 ± 5.38
	Malvidin	273.56 ± 3.81
Un-extractable hawthorn polyphenols	Catechin	27.22 ± 1.46
	chlorogenic acid	48.92 ± 1.28
	Caffeic acid	31.83 ± 0.45
	Gallic acid	29.66 ± 0.83
	Hyperoside	68.41 ± 1.25
	Isoquercitrin	80.87 ± 0.88
	Quercetin	96.69 ± 12.58
	Epicatechin	71.17 ± 1.76
	Phlorizin	55.20 ± 0.66
	Rutin	75.12 ± 3.18
	Ferulic acid	90.23 ± 0.12
	Protocatechuic acid	52.31 ± 0.14

Supplementary Table 2 Evaluation criteria for the appearance and taste of the tested fruits during storage

Score	Appearance assessment	Taste assessment
1	An overall green color, or large areas of decay and shrinkage appear on the surface.	Absence of fruity aroma, astringent flesh, excessive sourness, or off-flavors.
2	Greenish coloration, surface shrinkage, minor brown spots, or indentations.	Absence of fruity fragrance, slight astringency and sourness of the flesh, and no detectable off-odors.
3	Dull color, no decay, slight shriveling on the surface.	Faint distinctive aroma, non-astringent flesh, slight sweetness.
4	Reasonably uniform color, fairly bright appearance, plump shape, no decay.	A lighter distinctive aroma, sweet, fresh, delicious, and non-astringent flesh.
5	Uniformly bright color, fresh and bright surface, plump shape, no decay.	Rich distinctive aroma, sweet, fresh, delicious, and non-astringent flesh.

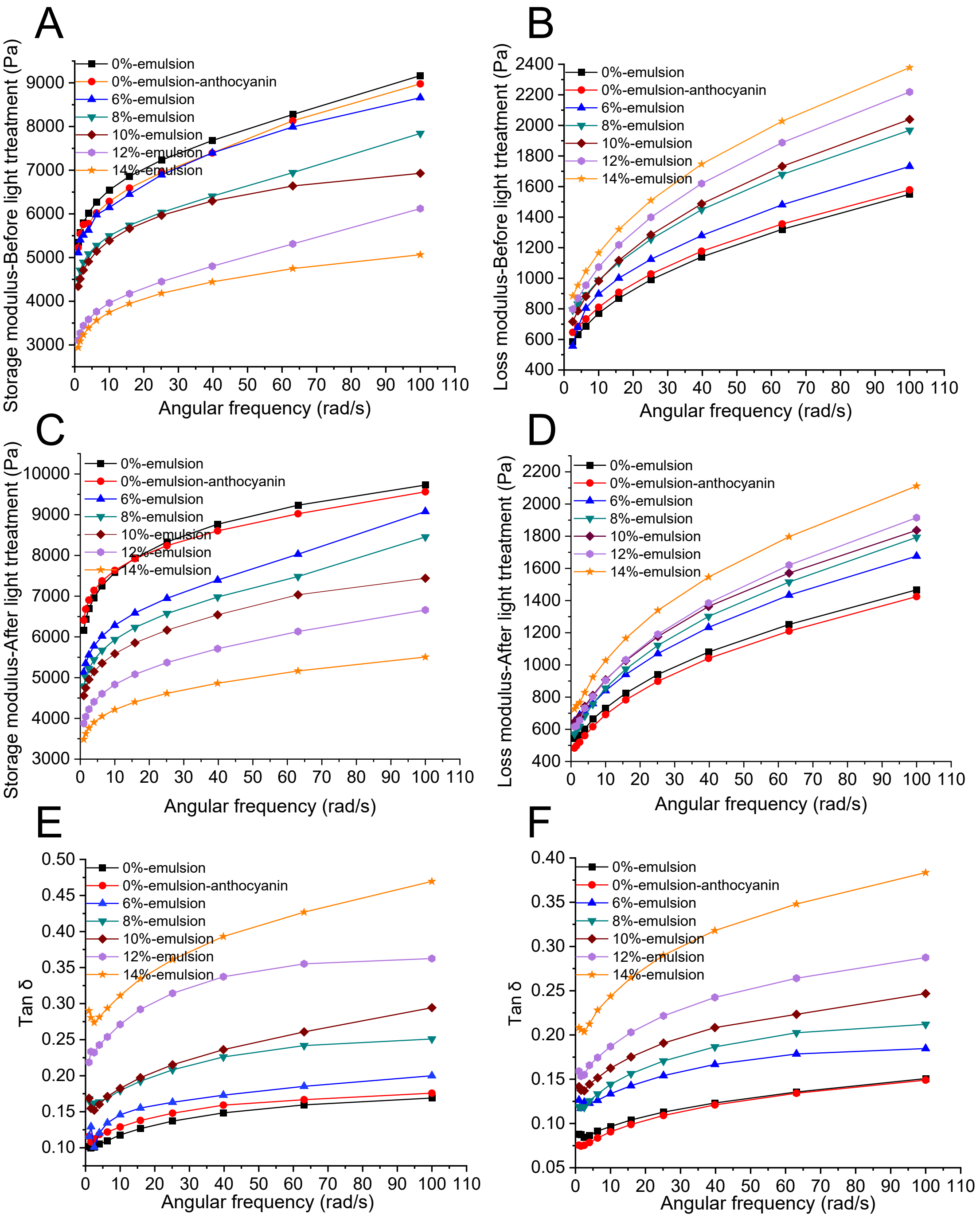

Supplementary Fig. 1 Frequency scanning of printed inks with varying essential oil emulsion contents. **A** Storage modulus before light exposure. **B** Loss modulus before light exposure. **C** Storage modulus after light exposure. D Loss modulus after light exposure. **E** Tanδ before light exposure. **F** Tanδ after light exposure. n = 3 independent samples per group. Source data are provided as a Source Data file.

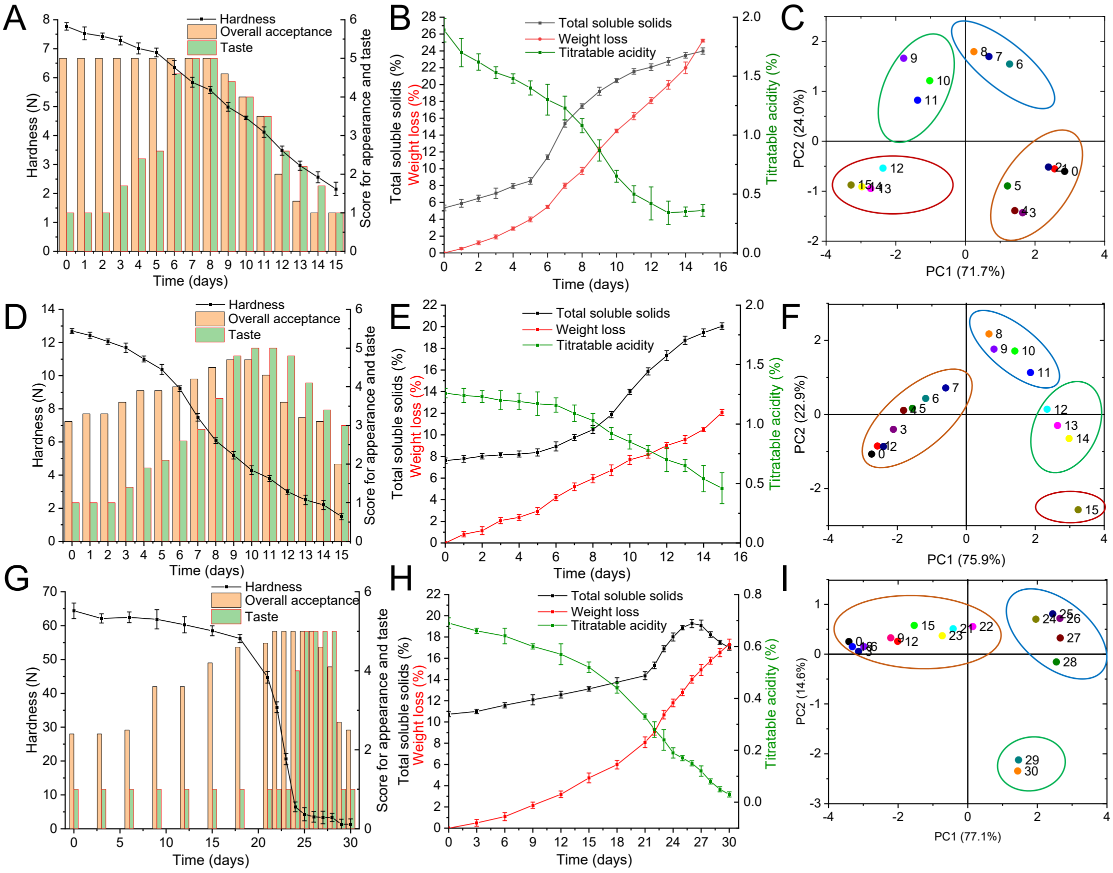

Supplementary Fig. 2 Quality changes in respiring climacteric fruits. Changes in the hardness, taste, and overall acceptance of **A** kiwi fruits, **D** green mangoes, and **G** persimmons during storage at 25 °C. Changes in the total soluble solids, weight loss, and titratable acidity of **B** kiwi fruits, **E** green mangoes, and **H** persimmons during storage at 25 °C. Discrimination of quality grades of **C** kiwi fruits, **F** green mangoes, and **I** persimmons using principal component analysis. All data are mean ± S.D. n = 3 independent samples per group. Source data are provided as a Source Data file.

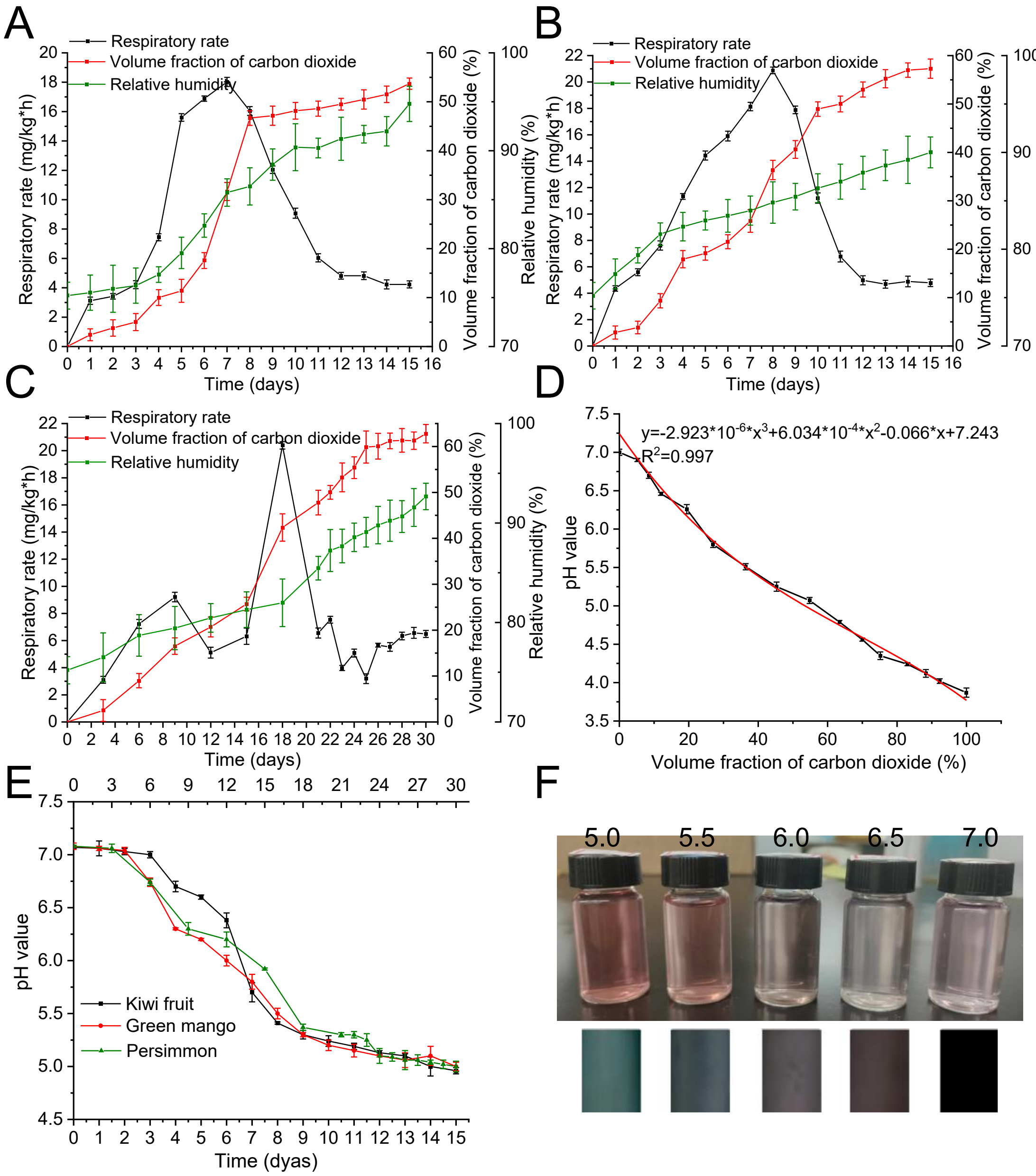

Supplementary Fig. 3 Changes in the internal environment of packaging containing respiring climacteric fruits during storage at 25 °C. A Changes in the respiration rate of kiwi fruits, and variations in carbon dioxide levels and humidity within the plastic box. **B** Changes in the respiration rate of green mangoes, and variations in carbon dioxide levels and humidity within the plastic box. **C** Changes in the respiration rate of persimmons, and variations in carbon dioxide levels and humidity within the plastic box. **D** Simulation of pH value corresponding to the different volume of carbon dioxide. **E** Changes in pH value caused by carbon dioxide content in the plastic box. **F** Color changes of the indicator at different pH values, and RGB images of the indicator's color at different pH values compared with its color at pH = 7.0. All data are mean ± S.D. n = 3 independent samples per group. Source data of Supplementary Fig. 4A-E are provided as a Source Data file.

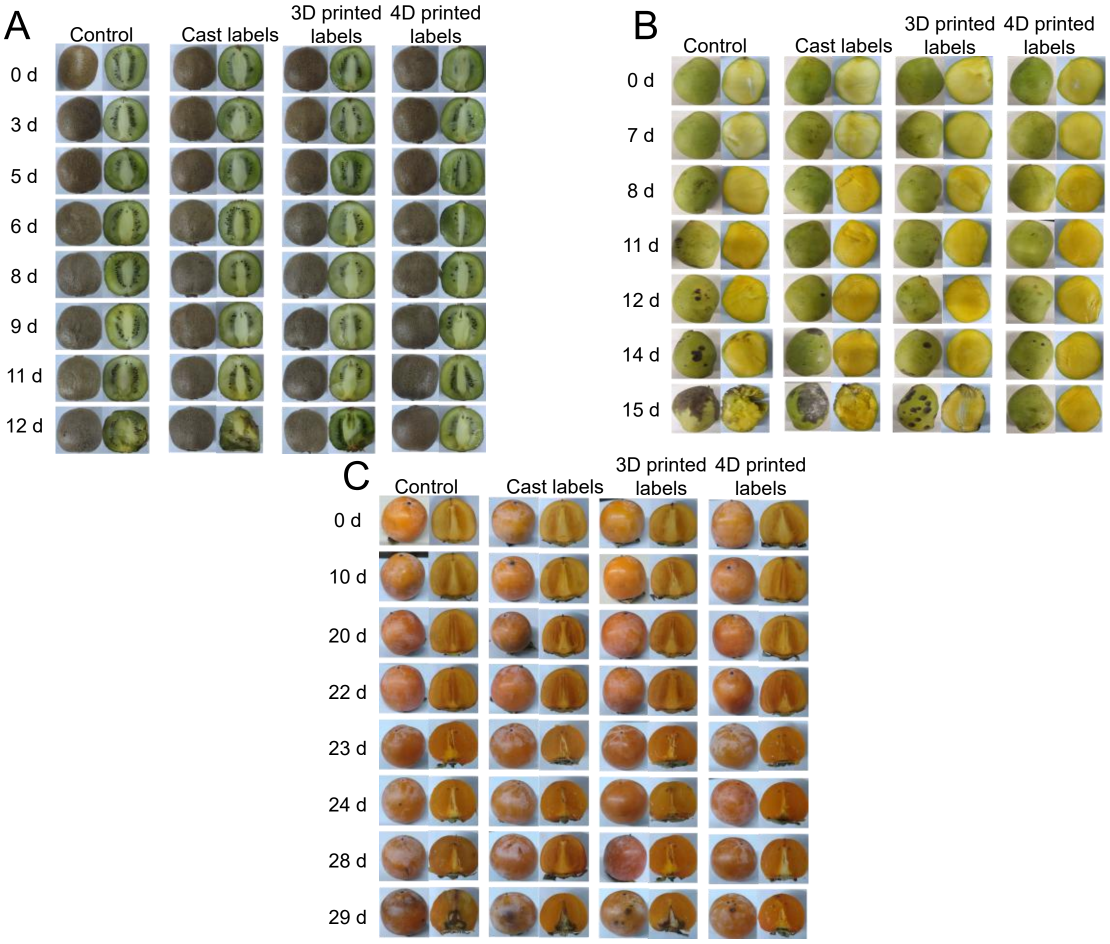

Supplementary Fig. 4 Macroscopic quality changes in respiring climacteric fruits during storage at 25 °C. A kiwi fruits. **B** Green mangoes. **C** persimmons. n = 3 independent samples per group.

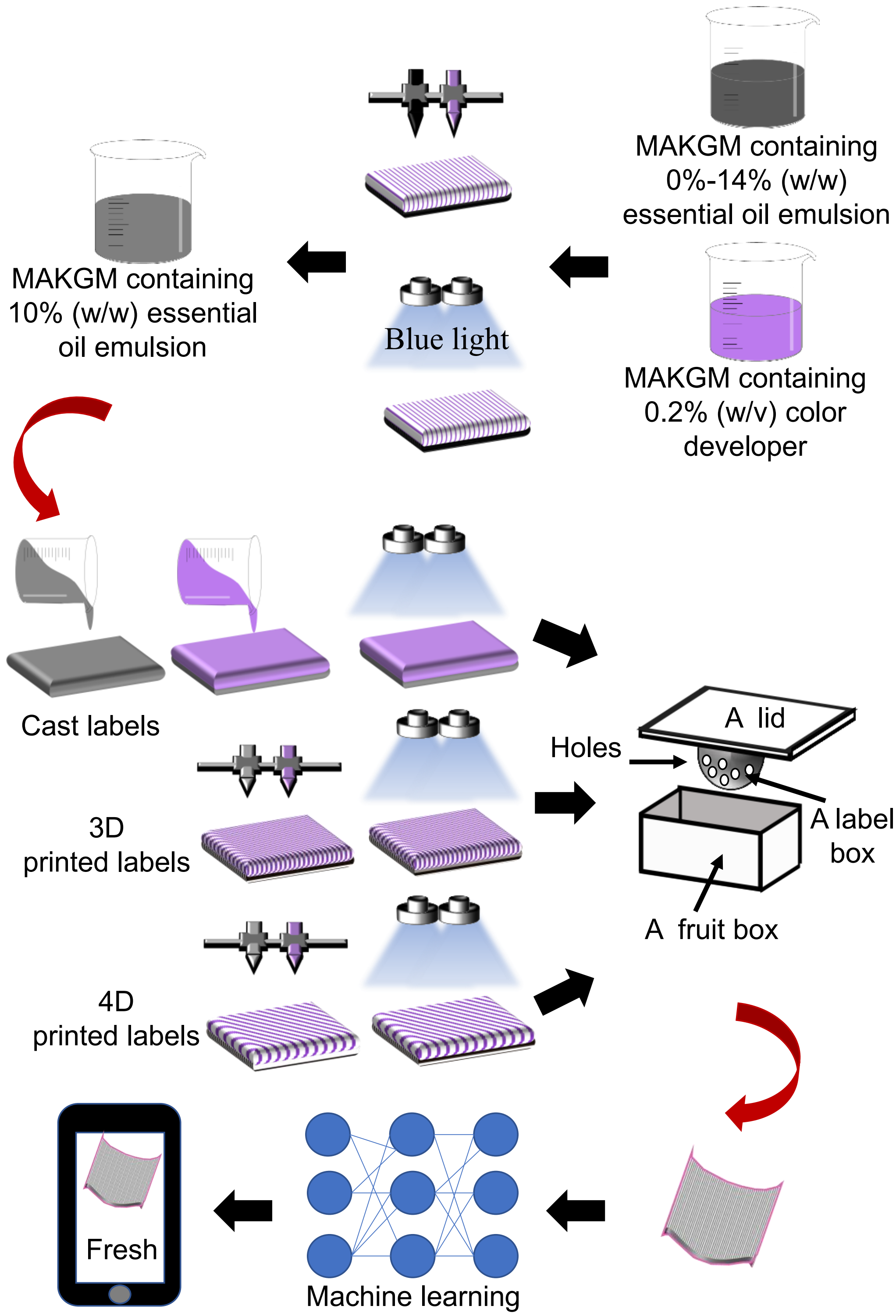

Supplementary Fig. 5 Schematic diagram of the preparation and application process for cast and printed labels.

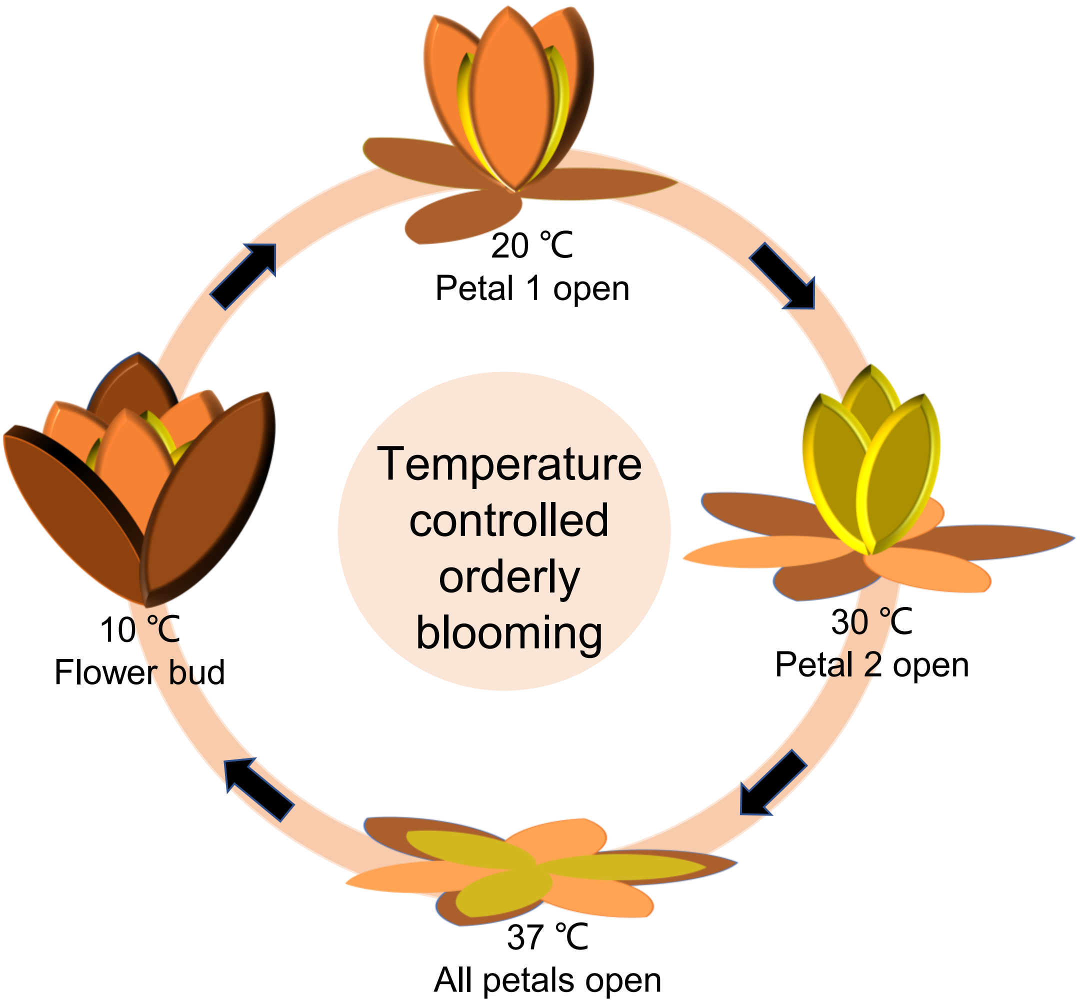

Supplementary Fig. 6 The design diagram of the flower-shaped pH-temperature responsive label.

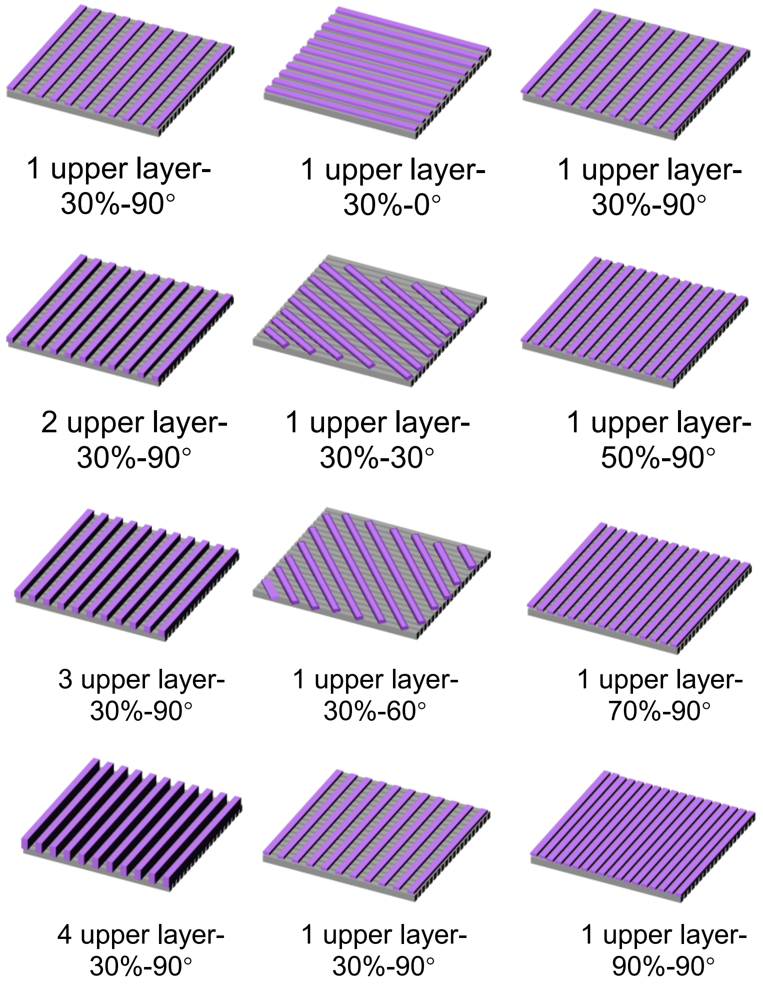

Supplementary Fig. 7 Schematic diagrams of different structural designs for 4D printed labels.
