# Supplementary material for: 4D printed deformation labels with machine learning for monitoring and preservation of respiring climacteric fruits
Source: Nat Commun. 2025 Nov 21;16:11525. doi: 10.1038/s41467-025-66554-6 (PMC12749378; doi:10.1038/s41467-025-66554-6)
Supplement: Supplementary file 2 — Description of Additional Supplementary Files [file 41467_2025_66554_MOESM2_ESM.pdf]

Description of Additional Supplementary Files

Supplementary Video: demonstrates the deformation and color change of 4D printed labels under the simulated conditions. The label consists of an upper layer made of modified konjac glucomannan loaded with anthocyanins, and a lower layer made of modified konjac glucomannan loaded with a 10% (w/w) essential oil emulsion. The upper layer is printed as a single layer with a 30% infill ratio, while the lower layer has a 100% infill ratio and a 90° extrusion path alternating angle. The specific procedure involves placing the 4D printed label in a phosphate buffer solution with a pH of 4 and observing its sensory and shape changes. During observation, the label was found to curl inward until reaching its maximum curvature. As the label continued to absorb water and expand, the curled structure began to unfurl. Throughout this process, the color of the label changed from purple to red.

Supplementary Code: contains the model code for processing, training, and evaluating images of 4D printed labels, along with the necessary environment setup and usage instructions. The optimal model, selected based on training and evaluation accuracy, is capable of determining the freshness level of respiring climacteric fruits by analyzing images that capture the label's changes in color and shape.
