# Supplementary material for: 4D printed deformation labels with machine learning for monitoring and preservation of respiring climacteric fruits
Source: Nat Commun. 2025 Nov 21;16:11525. doi: 10.1038/s41467-025-66554-6 (PMC12749378; doi:10.1038/s41467-025-66554-6)
Supplement: Supplementary file 4 — Supplementary Code [file 41467_2025_66554_MOESM4_ESM.zip › Supplementary Code/Code-classification.pdf]

```python
import matplotlib.pyplot as plt
import numpy as np
import torch
from torch import nn

from nets import get_model_from_name
from utils.utils import (cvtColor, get_classes, letterbox_image,
                         preprocess_input)


#--------------------------------------------#
#   使用自己训练好的模型预测需要修改 3 个参数
#   model_path 和 classes_path 和 backbone 都需要修改!
#--------------------------------------------#
class Classification(object):
    _defaults = {
        #--------------------------------------------------------------------------
-------------#
        #   使用自己训练好的模型进行预测一定要修改 model_path 和 classes_path!
        #   model_path 指向 logs 文件夹下的权值文件，classes_path 指向
model_data 下的 txt
        #   如果出现 shape 不匹配，同时要注意训练时的 model_path 和
classes_path 参数的修改
        #--------------------------------------------------------------------------
-------------#
        "model_path"        : 'logs/马头兰-ghostnet/ep774-loss0.341-
val_loss0.355.pth',
        "classes_path"      : 'model_data/cls_classes.txt',
        #--------------------------------------------------------------------
--------#
        #   输入的图片大小
        #--------------------------------------------------------------------
--------#
        "input_shape"       : [224, 224],
        #--------------------------------------------------------------------
--------#
        #   所用模型种类:
        #   mobilenet、resnet50、vgg16、vit
        #--------------------------------------------------------------------
--------#
        "backbone"          : 'ghostnet',
        #--------------------------------------------------------------------
--------#
        #   该变量用于控制是否使用 letterbox_image 对输入图像进行不失真的 resize
```

```
        #   否则对图像进行 CenterCrop
        #---------------------------------------------------------------------
-------#
        "letterbox_image"   : True,
        #--------------------------------#
        #   是否使用 Cuda
        #   没有 GPU 可以设置成 False
        #--------------------------------#
        "cuda"              : True
    }

    @classmethod
    def get_defaults(cls, n):
        if n in cls._defaults:
            return cls._defaults[n]
        else:
            return "Unrecognized attribute name '" + n + "'"

    #---------------------------------------------------#
    #   初始化 classification
    #---------------------------------------------------#
    def __init__(self, **kwargs):
        self.__dict__.update(self._defaults)
        for name, value in kwargs.items():
            setattr(self, name, value)

        #---------------------------------------------------#
        #   获得种类
        #---------------------------------------------------#
        self.class_names, self.num_classes =
get_classes(self.classes_path)
        self.generate()

    #---------------------------------------------------#
    #   获得所有的分类
    #---------------------------------------------------#
    def generate(self):
        #---------------------------------------------------#
        #   载入模型与权值
        #---------------------------------------------------#
        if self.backbone == "vit":
            self.model  =
get_model_from_name[self.backbone](input_shape=self.input_shape,
num_classes=self.num_classes, pretrained=self.pretrained)
```

```python
        elif self.backbone == "shufflenet_v2" or self.backbone ==
"ghostnet":
            self.model =
get_model_from_name[self.backbone](num_classes=self.num_classes)
        else:
            self.model  =
get_model_from_name[self.backbone](num_classes = self.num_classes,
pretrained = False)
        '''if self.backbone != "vit":
            self.model  =
get_model_from_name[self.backbone](num_classes = self.num_classes,
pretrained = False)
        else:
            self.model  =
get_model_from_name[self.backbone](input_shape = self.input_shape,
num_classes = self.num_classes, pretrained = False)'''
        device      = torch.device('cuda' if torch.cuda.is_available()
else 'cpu')
        self.model.load_state_dict(torch.load(self.model_path,
map_location=device))
        self.model  = self.model.eval()
        print('{} model, and classes loaded.'.format(self.model_path))

        if self.cuda:
            self.model = nn.DataParallel(self.model)
            self.model = self.model.cuda()

    #---------------------------------------------------#
    #   检测图片
    #---------------------------------------------------#
    def detect_image(self, image):
        #---------------------------------------------------------#
        #   在这里将图像转换成RGB图像，防止灰度图在预测时报错。
        #   代码仅仅支持RGB图像的预测，所有其它类型的图像都会转化成RGB
        #---------------------------------------------------------#
        image       = cvtColor(image)
        #---------------------------------------------------#
        #   对图片进行不失真的resize
        #---------------------------------------------------#
        image_data  = letterbox_image(image, [self.input_shape[1],
self.input_shape[0]], self.letterbox_image)
        #---------------------------------------------------------#
        #   归一化+添加上batch_size维度+转置
        #---------------------------------------------------------#
```

```
        image_data  = 
np.transpose(np.expand_dims(preprocess_input(np.array(image_data, 
np.float32)), 0), (0, 3, 1, 2))

        with torch.no_grad():
            photo   = torch.from_numpy(image_data)
            if self.cuda:
                photo = photo.cuda()
            #---------------------------------------------------#
            #   图片传入网络进行预测
            #---------------------------------------------------#
            preds   = torch.softmax(self.model(photo)[0], dim=-
1).cpu().numpy()
        #---------------------------------------------------#
        #   获得所属种类
        #---------------------------------------------------#
        class_name  = self.class_names[np.argmax(preds)]
        probability = np.max(preds)

        #---------------------------------------------------#
        #   绘图并写字
        #---------------------------------------------------#
        plt.subplot(1, 1, 1)
        plt.imshow(np.array(image))
        plt.title('Class:%s Probability:%.3f' %(class_name, 
probability))
        plt.show()
        return class_name
```
