# Supplementary material for: 4D printed deformation labels with machine learning for monitoring and preservation of respiring climacteric fruits
Source: Nat Commun. 2025 Nov 21;16:11525. doi: 10.1038/s41467-025-66554-6 (PMC12749378; doi:10.1038/s41467-025-66554-6)
Supplement: Supplementary file 4 — Supplementary Code [file 41467_2025_66554_MOESM4_ESM.zip › Supplementary Code/Code-eval.pdf]

```python
import os

import numpy as np
import torch

from classification import (Classification, cvtColor,
letterbox_image,
                            preprocess_input)
from utils.utils import letterbox_image
from utils.utils_metrics import evaluteTop1_5

#------------------------------------------------------#
#   test_annotation_path    测试图片路径和标签
#------------------------------------------------------#
test_annotation_path    = 'cls_test.txt'
#------------------------------------------------------#
#   metrics_out_path        指标保存的文件夹
#------------------------------------------------------#
metrics_out_path        = "metrics_out"

class Eval_Classification(Classification):
    def detect_image(self, image):
        #---------------------------------------------------------#
        #   在这里将图像转换成 RGB 图像，防止灰度图在预测时报错。
        #   代码仅仅支持 RGB 图像的预测，所有其它类型的图像都会转化成 RGB
        #---------------------------------------------------------#
        image       = cvtColor(image)
        #---------------------------------------------------#
        #   对图片进行不失真的 resize
        #---------------------------------------------------#
        image_data  = letterbox_image(image, [self.input_shape[1],
self.input_shape[0]], self.letterbox_image)
        #---------------------------------------------------------#
        #   归一化+添加上 batch_size 维度+转置
        #---------------------------------------------------------#
        image_data  =
np.transpose(np.expand_dims(preprocess_input(np.array(image_data,
np.float32)), 0), (0, 3, 1, 2))

        with torch.no_grad():
            photo   =
torch.from_numpy(image_data).type(torch.FloatTensor)
            if self.cuda:
                photo = photo.cuda()
```

```
            #--------------------------------------------------#
            #   图片传入网络进行预测
            #--------------------------------------------------#
            preds   = torch.softmax(self.model(photo)[0], dim=-
1).cpu().numpy()

        return preds

if __name__ == "__main__":
    if not os.path.exists(metrics_out_path):
        os.makedirs(metrics_out_path)

    classfication = Eval_Classification()

    with open("./cls_test.txt","r") as f:
        lines = f.readlines()
    top1, top5, Recall, Precision = evaluteTop1_5(classfication,
lines, metrics_out_path)
    print("top-1 accuracy = %.2f%%" % (top1*100))
    print("top-5 accuracy = %.2f%%" % (top5*100))
    print("mean Recall = %.2f%%" % (np.mean(Recall)*100))
    print("mean Precision = %.2f%%" % (np.mean(Precision)*100))
```
