# Supplementary material for: 4D printed deformation labels with machine learning for monitoring and preservation of respiring climacteric fruits
Source: Nat Commun. 2025 Nov 21;16:11525. doi: 10.1038/s41467-025-66554-6 (PMC12749378; doi:10.1038/s41467-025-66554-6)
Supplement: Supplementary file 4 — Supplementary Code [file 41467_2025_66554_MOESM4_ESM.zip › Supplementary Code/Code-Ghostnet.pdf]

```python
import torch
import torch.nn as nn
import torch.nn.functional as F
import math
from torch.hub import load_state_dict_from_url
model_urls = {
    'mobilenet_v1': 'https://drive.google.com/drive/folders/1pKn-
RifvJGWiOx0ZCRLtCXM5GT5lAluu?usp=sharing',
}
__all__ = ['ghostnet']


def _make_divisible(v, divisor, min_value=None):
    """
    This function is taken from the original tf repo.
    It ensures that all layers have a channel number that is
divisible by 8
    It can be seen here:

https://github.com/tensorflow/models/blob/master/research/slim/nets/m
obilenet/mobilenet.py
    """
    if min_value is None:
        min_value = divisor
    new_v = max(min_value, int(v + divisor / 2) // divisor * divisor)
    # Make sure that round down does not go down by more than 10%.
    if new_v < 0.9 * v:
        new_v += divisor
    return new_v


def hard_sigmoid(x, inplace: bool = False):
    if inplace:
        return x.add_(3.).clamp_(0., 6.).div_(6.)
    else:
        return F.relu6(x + 3.) / 6.


class SqueezeExcite(nn.Module):
    def __init__(self, in_chs, se_ratio=0.25, reduced_base_chs=None,
                 act_layer=nn.ReLU, gate_fn=hard_sigmoid, divisor=4,
**_):
        super(SqueezeExcite, self).__init__()
        self.gate_fn = gate_fn
```

```
        reduced_chs = _make_divisible((reduced_base_chs or in_chs) *
se_ratio, divisor)
        self.avg_pool = nn.AdaptiveAvgPool2d(1)
        self.conv_reduce = nn.Conv2d(in_chs, reduced_chs, 1,
bias=True)
        self.act1 = act_layer(inplace=True)
        self.conv_expand = nn.Conv2d(reduced_chs, in_chs, 1,
bias=True)

    def forward(self, x):
        x_se = self.avg_pool(x)
        x_se = self.conv_reduce(x_se)
        x_se = self.act1(x_se)
        x_se = self.conv_expand(x_se)
        x = x * self.gate_fn(x_se)
        return x


class ConvBnAct(nn.Module):
    def __init__(self, in_chs, out_chs, kernel_size,
                 stride=1, act_layer=nn.ReLU):
        super(ConvBnAct, self).__init__()
        self.conv = nn.Conv2d(in_chs, out_chs, kernel_size, stride,
kernel_size // 2, bias=False)
        self.bn1 = nn.BatchNorm2d(out_chs)
        self.act1 = act_layer(inplace=True)

    def forward(self, x):
        x = self.conv(x)
        x = self.bn1(x)
        x = self.act1(x)
        return x


class GhostModule(nn.Module):
    def __init__(self, inp, oup, kernel_size=1, ratio=2, dw_size=3,
stride=1, relu=True):
        super(GhostModule, self).__init__()
        self.oup = oup
        init_channels = math.ceil(oup / ratio)
        new_channels = init_channels * (ratio - 1)

        self.primary_conv = nn.Sequential(
            nn.Conv2d(inp, init_channels, kernel_size, stride,
```

```
kernel_size // 2, bias=False),
            nn.BatchNorm2d(init_channels),
            nn.ReLU(inplace=True) if relu else nn.Sequential(),
        )

        self.cheap_operation = nn.Sequential(
            nn.Conv2d(init_channels, new_channels, dw_size, 1, dw_size
// 2, groups=init_channels, bias=False),
            nn.BatchNorm2d(new_channels),
            nn.ReLU(inplace=True) if relu else nn.Sequential(),
        )

    def forward(self, x):
        x1 = self.primary_conv(x)
        x2 = self.cheap_operation(x1)
        out = torch.cat([x1, x2], dim=1)
        return out[:, :self.oup, :, :]


class GhostBottleneck(nn.Module):
    """ Ghost bottleneck w/ optional SE"""

    def __init__(self, in_chs, mid_chs, out_chs, dw_kernel_size=3,
                 stride=1, act_layer=nn.ReLU, se_ratio=0.):
        super(GhostBottleneck, self).__init__()
        has_se = se_ratio is not None and se_ratio > 0.
        self.stride = stride

        # Point-wise expansion
        self.ghost1 = GhostModule(in_chs, mid_chs, relu=True)

        # Depth-wise convolution
        if self.stride > 1:
            self.conv_dw = nn.Conv2d(mid_chs, mid_chs, dw_kernel_size,
stride=stride,
                                     padding=(dw_kernel_size - 1) // 2,
                                     groups=mid_chs, bias=False)
            self.bn_dw = nn.BatchNorm2d(mid_chs)

        # Squeeze-and-excitation
        if has_se:
            self.se = SqueezeExcite(mid_chs, se_ratio=se_ratio)
        else:
            self.se = None
```

```
        # Point-wise linear projection
        self.ghost2 = GhostModule(mid_chs, out_chs, relu=False)

        # shortcut
        if (in_chs == out_chs and self.stride == 1):
            self.shortcut = nn.Sequential()
        else:
            self.shortcut = nn.Sequential(
                nn.Conv2d(in_chs, in_chs, dw_kernel_size,
stride=stride,
                       padding=(dw_kernel_size - 1) // 2,
groups=in_chs, bias=False),
                nn.BatchNorm2d(in_chs),
                nn.Conv2d(in_chs, out_chs, 1, stride=1, padding=0,
bias=False),
                nn.BatchNorm2d(out_chs),
            )

    def forward(self, x):
        residual = x

        # 1st ghost bottleneck
        x = self.ghost1(x)

        # Depth-wise convolution
        if self.stride > 1:
            x = self.conv_dw(x)
            x = self.bn_dw(x)

        # Squeeze-and-excitation
        if self.se is not None:
            x = self.se(x)

        # 2nd ghost bottleneck
        x = self.ghost2(x)

        x += self.shortcut(residual)
        return x


class GhostNet(nn.Module):
    def __init__(self, cfgs, num_classes=1000, width=1.0,
dropout=0.2):
```

```
        super(GhostNet, self).__init__()
        # setting of inverted residual blocks
        self.cfgs = cfgs
        self.dropout = dropout

        # building first layer
        output_channel = _make_divisible(16 * width, 4)
        self.conv_stem = nn.Conv2d(3, output_channel, 3, 2, 1,
bias=False)
        self.bn1 = nn.BatchNorm2d(output_channel)
        self.act1 = nn.ReLU(inplace=True)
        input_channel = output_channel

        # building inverted residual blocks
        stages = []
        block = GhostBottleneck
        for cfg in self.cfgs:
            layers = []
            for k, exp_size, c, se_ratio, s in cfg:
                output_channel = _make_divisible(c * width, 4)
                hidden_channel = _make_divisible(exp_size * width, 4)
                layers.append(block(input_channel, hidden_channel,
output_channel, k, s,
                                    se_ratio=se_ratio))
                input_channel = output_channel
            stages.append(nn.Sequential(*layers))

        output_channel = _make_divisible(exp_size * width, 4)
        stages.append(nn.Sequential(ConvBnAct(input_channel,
output_channel, 1)))
        input_channel = output_channel

        self.blocks = nn.Sequential(*stages)

        # building last several layers
        output_channel = 1280
        self.global_pool = nn.AdaptiveAvgPool2d((1, 1))
        self.conv_head = nn.Conv2d(input_channel, output_channel, 1,
1, 0, bias=True)
        self.act2 = nn.ReLU(inplace=True)
        self.classifier = nn.Linear(output_channel, num_classes)

    def forward(self, x):
        x = self.conv_stem(x)
```

```
        x = self.bn1(x)
        x = self.act1(x)
        x = self.blocks(x)
        x = self.global_pool(x)
        x = self.conv_head(x)
        x = self.act2(x)
        x = x.view(x.size(0), -1)
        if self.dropout > 0.:
            x = F.dropout(x, p=self.dropout, training=self.training)
        x = self.classifier(x)
        return x


def ghostnet(**kwargs):
    """
    Constructs a GhostNet model
    """
    cfgs = [
        # k, t, c, SE, s
        # stage1
        [[3, 16, 16, 0, 1]],
        # stage2
        [[3, 48, 24, 0, 2]],
        [[3, 72, 24, 0, 1]],
        # stage3
        [[5, 72, 40, 0.25, 2]],
        [[5, 120, 40, 0.25, 1]],
        # stage4
        [[3, 240, 80, 0, 2]],
        [[3, 200, 80, 0, 1],
         [3, 184, 80, 0, 1],
         [3, 184, 80, 0, 1],
         [3, 480, 112, 0.25, 1],
         [3, 672, 112, 0.25, 1]
         ],
        # stage5
        [[5, 672, 160, 0.25, 2]],
        [[5, 960, 160, 0, 1],
         [5, 960, 160, 0.25, 1],
         [5, 960, 160, 0, 1],
         [5, 960, 160, 0.25, 1]
         ]
    ]
    return GhostNet(cfgs, **kwargs)
```
