# Supplementary material for: 4D printed deformation labels with machine learning for monitoring and preservation of respiring climacteric fruits
Source: Nat Commun. 2025 Nov 21;16:11525. doi: 10.1038/s41467-025-66554-6 (PMC12749378; doi:10.1038/s41467-025-66554-6)
Supplement: Supplementary file 4 — Supplementary Code [file 41467_2025_66554_MOESM4_ESM.zip › Supplementary Code/Code-Mobilenet.pdf]

```python
from torch import nn
# from torchvision.models.utils import load_state_dict_from_url
from torch.hub import load_state_dict_from_url

__all__ = ['MobileNetV2', 'mobilenet_v2']


model_urls = {
    'mobilenet_v2':
'https://download.pytorch.org/models/mobilenet_v2-b0353104.pth',
}


def _make_divisible(v, divisor, min_value=None):
    if min_value is None:
        min_value = divisor
    new_v = max(min_value, int(v + divisor / 2) // divisor * divisor)
    if new_v < 0.9 * v:
        new_v += divisor
    return new_v

class ConvBNReLU(nn.Sequential):
    def __init__(self, in_planes, out_planes, kernel_size=3,
stride=1, groups=1):
        padding = (kernel_size - 1) // 2
        super(ConvBNReLU, self).__init__(
            nn.Conv2d(in_planes, out_planes, kernel_size, stride,
padding, groups=groups, bias=False),
            nn.BatchNorm2d(out_planes),
            nn.ReLU6(inplace=True)
        )

class InvertedResidual(nn.Module):
    def __init__(self, inp, oup, stride, expand_ratio):
        super(InvertedResidual, self).__init__()
        self.stride = stride
        assert stride in [1, 2]

        hidden_dim = int(round(inp * expand_ratio))
        self.use_res_connect = self.stride == 1 and inp == oup

        layers = []
        if expand_ratio != 1:
            layers.append(ConvBNReLU(inp, hidden_dim, kernel_size=1))
```

```python
        layers.extend([
            ConvBNReLU(hidden_dim, hidden_dim, stride=stride,
groups=hidden_dim),
            nn.Conv2d(hidden_dim, oup, 1, 1, 0, bias=False),
            nn.BatchNorm2d(oup),
        ])
        self.conv = nn.Sequential(*layers)

    def forward(self, x):
        if self.use_res_connect:
            return x + self.conv(x)
        else:
            return self.conv(x)


class MobileNetV2(nn.Module):
    def __init__(self, num_classes=1000, width_mult=1.0,
inverted_residual_setting=None, round_nearest=8):
        super(MobileNetV2, self).__init__()
        block = InvertedResidual
        input_channel = 32
        last_channel = 1280

        if inverted_residual_setting is None:
            inverted_residual_setting = [
                # t, c, n, s
                # 112, 112, 32 -> 112, 112, 16
                [1, 16, 1, 1],
                # 112, 112, 16 -> 56, 56, 24
                [6, 24, 2, 2],
                # 56, 56, 24 -> 28, 28, 32
                [6, 32, 3, 2],
                # 28, 28, 32 -> 14, 14, 64
                [6, 64, 4, 2],
                # 14, 14, 64 -> 14, 14, 96
                [6, 96, 3, 1],
                # 14, 14, 96 -> 7, 7, 160
                [6, 160, 3, 2],
                # 7, 7, 160 -> 7, 7, 320
                [6, 320, 1, 1],
            ]

        if len(inverted_residual_setting) == 0 or
len(inverted_residual_setting[0]) != 4:
```

```
            raise ValueError("inverted_residual_setting should be non-
empty "
                             "or a 4-element list, got
{}".format(inverted_residual_setting))

        input_channel = _make_divisible(input_channel * width_mult,
round_nearest)
        self.last_channel = _make_divisible(last_channel * max(1.0,
width_mult), round_nearest)

        # 224, 224, 3 -> 112, 112, 32
        features = [ConvBNReLU(3, input_channel, stride=2)]

        for t, c, n, s in inverted_residual_setting:
            output_channel = _make_divisible(c * width_mult,
round_nearest)
            for i in range(n):
                stride = s if i == 0 else 1
                features.append(block(input_channel, output_channel,
stride, expand_ratio=t))
                input_channel = output_channel

        # 7, 7, 320 -> 7,7,1280
        features.append(ConvBNReLU(input_channel, self.last_channel,
kernel_size=1))
        self.features = nn.Sequential(*features)

        self.classifier = nn.Sequential(
            nn.Dropout(0.2),
            nn.Linear(self.last_channel, num_classes),
        )

        for m in self.modules():
            if isinstance(m, nn.Conv2d):
                nn.init.kaiming_normal_(m.weight, mode='fan_out')
                if m.bias is not None:
                    nn.init.zeros_(m.bias)
            elif isinstance(m, nn.BatchNorm2d):
                nn.init.ones_(m.weight)
                nn.init.zeros_(m.bias)
            elif isinstance(m, nn.Linear):
                nn.init.normal_(m.weight, 0, 0.01)
                nn.init.zeros_(m.bias)
```

```
    def forward(self, x):
        x = self.features(x)
        # 1280
        x = x.mean([2, 3])
        x = self.classifier(x)
        return x

    def freeze_backbone(self):
        for param in self.features.parameters():
            param.requires_grad = False

    def Unfreeze_backbone(self):
        for param in self.features.parameters():
            param.requires_grad = True


def mobilenet_v2(pretrained=False, progress=True, num_classes=1000):
    model = MobileNetV2()
    if pretrained:
        state_dict =
load_state_dict_from_url(model_urls['mobilenet_v2'],
model_dir='./model_data',
                                              progress=progress)
        model.load_state_dict(state_dict)

    if num_classes!=1000:
        model.classifier = nn.Sequential(
                nn.Dropout(0.2),
                nn.Linear(model.last_channel, num_classes),
            )
    return model
```
