# Supplementary material for: 4D printed deformation labels with machine learning for monitoring and preservation of respiring climacteric fruits
Source: Nat Commun. 2025 Nov 21;16:11525. doi: 10.1038/s41467-025-66554-6 (PMC12749378; doi:10.1038/s41467-025-66554-6)
Supplement: Supplementary file 4 — Supplementary Code [file 41467_2025_66554_MOESM4_ESM.zip › Supplementary Code/Code-README.pdf]

README

Section	Content description	Example description
Brief introduction	This code is designed to predict fruit freshness by analyzing the dual changes in both color and shape of 4D printed labels inside the fruit packaging.	This zip file includes the source code and datasets employed in this study for the training and evaluation of fruit freshness.
System requirements	This section lists the required environment and dependencies for running the application.	1. Operating system: Linux, Windows 10+<br>2. Programming language: Python 3.8+<br>3. Dependencies: Torch, NumPy, Pandas (See requirements.pdf for full list)
Installation guide	The process involves obtaining and setting up the program.	1. Download and unzip the package.<br>2. Install the dependencies: pip install-r requirements.pdf
File structure	This section explains the function of the key files and directories.	1. nets: Neural network model architecture code<br>2. train.pdf: Code for training the model<br>3. eval.pdf: Code for evaluating the model<br>4. classification.pdf: Code to generate training and test datasets<br>5. README.pdf: Project overview and documentation (This file)<br>6. requirements.pdf: List of Python dependencies
Operating instructions	This section describes how to run the program.	1. Training and test set generation: classification.pdf<br>2. Model training: train.pdf<br>3. Evaluation: eval.pdf
Common issues	This section lists potential problems and their solutions.	1. If you get a prompt about a missing module when running, please make sure to run pip install-r requirements.pdf.<br>2. To change the training model, please modify the backbone defined in train.pdf.
Contact information	Here you can find the contact details of the author or maintainer.	E-mail: Tengxx116587@163.com
