# Supplementary material for: 4D printed deformation labels with machine learning for monitoring and preservation of respiring climacteric fruits
Source: Nat Commun. 2025 Nov 21;16:11525. doi: 10.1038/s41467-025-66554-6 (PMC12749378; doi:10.1038/s41467-025-66554-6)
Supplement: Supplementary file 4 — Supplementary Code [file 41467_2025_66554_MOESM4_ESM.zip › Supplementary Code/Code-requirements.pdf]

```
scipy==1.2.1
numpy==1.17.0
matplotlib==3.1.2
opencv_python==4.1.2.30
torch==1.2.0
torchvision==0.4.0
tqdm==4.60.0
Pillow==8.2.0
h5py==2.10.0
```
