# Supplementary material for: 4D printed deformation labels with machine learning for monitoring and preservation of respiring climacteric fruits
Source: Nat Commun. 2025 Nov 21;16:11525. doi: 10.1038/s41467-025-66554-6 (PMC12749378; doi:10.1038/s41467-025-66554-6)
Supplement: Supplementary file 4 — Supplementary Code [file 41467_2025_66554_MOESM4_ESM.zip › Supplementary Code/Code-Shufflenet.pdf]

```python
from collections import OrderedDict

import torch
import torch.nn as nn
import torch.nn.functional as F
from torch.nn import init


def _make_divisible(v, divisor, min_value=None):
    """
    This function is taken from the original tf repo.
    It ensures that all layers have a channel number that is
divisible by 8
    It can be seen here:

https://github.com/tensorflow/models/blob/master/research/slim/nets/m
obilenet/mobilenet.py
    :param v:
    :param divisor:
    :param min_value:
    :return:
    """
    if min_value is None:
        min_value = divisor
    new_v = max(min_value, int(v + divisor / 2) // divisor * divisor)
    # Make sure that round down does not go down by more than 10%.
    if new_v < 0.9 * v:
        new_v += divisor
    return new_v


def channel_shuffle(x, groups):
    batchsize, num_channels, height, width = x.data.size()
    assert (num_channels % groups == 0)
    channels_per_group = num_channels // groups
    # reshape
    x = x.view(batchsize, groups, channels_per_group, height, width)

    # transpose
    # - contiguous() required if transpose() is used before view().
    #   See https://github.com/pytorch/pytorch/issues/764
    x = torch.transpose(x, 1, 2).contiguous()

    # flatten
```

```python
    x = x.view(batchsize, -1, height, width)

    return x


class SELayer(nn.Module):
    def __init__(self, channel, reduction=16):
        super(SELayer, self).__init__()
        self.avg_pool = nn.AdaptiveAvgPool2d(1)
        self.fc = nn.Sequential(
            nn.Linear(channel, channel // reduction),
            nn.ReLU(inplace=True),
            nn.Linear(channel // reduction, channel),
            nn.Sigmoid()
        )

    def forward(self, x):
        b, c, _, _ = x.size()
        y = self.avg_pool(x).view(b, c)
        y = self.fc(y).view(b, c, 1, 1)
        return x * y


class BasicUnit(nn.Module):
    def __init__(self, inplanes, outplanes, c_tag=0.5,
activation=nn.ReLU, SE=False, residual=False, groups=2):
        super(BasicUnit, self).__init__()
        self.left_part = round(c_tag * inplanes)
        self.right_part_in = inplanes - self.left_part
        self.right_part_out = outplanes - self.left_part
        self.conv1 = nn.Conv2d(self.right_part_in,
self.right_part_out, kernel_size=1, bias=False)
        self.bn1 = nn.BatchNorm2d(self.right_part_out)
        self.conv2 = nn.Conv2d(self.right_part_out,
self.right_part_out, kernel_size=3, padding=1, bias=False,
                               groups=self.right_part_out)
        self.bn2 = nn.BatchNorm2d(self.right_part_out)
        self.conv3 = nn.Conv2d(self.right_part_out,
self.right_part_out, kernel_size=1, bias=False)
        self.bn3 = nn.BatchNorm2d(self.right_part_out)
        self.activation = activation(inplace=True)

        self.inplanes = inplanes
        self.outplanes = outplanes
```

```
        self.residual = residual
        self.groups = groups
        self.SE = SE
        if self.SE:
            self.SELayer = SELayer(self.right_part_out, 2)  # TODO

    def forward(self, x):
        left = x[:, :self.left_part, :, :]
        right = x[:, self.left_part:, :, :]
        out = self.conv1(right)
        out = self.bn1(out)
        out = self.activation(out)

        out = self.conv2(out)
        out = self.bn2(out)

        out = self.conv3(out)
        out = self.bn3(out)
        out = self.activation(out)

        if self.SE:
            out = self.SELayer(out)
        if self.residual and self.inplanes == self.outplanes:
            out += right

        return channel_shuffle(torch.cat((left, out), 1), self.groups)


class DownsampleUnit(nn.Module):
    def __init__(self, inplanes, c_tag=0.5, activation=nn.ReLU,
groups=2):
        super(DownsampleUnit, self).__init__()

        self.conv1r = nn.Conv2d(inplanes, inplanes, kernel_size=1,
bias=False)
        self.bn1r = nn.BatchNorm2d(inplanes)
        self.conv2r = nn.Conv2d(inplanes, inplanes, kernel_size=3,
stride=2, padding=1, bias=False, groups=inplanes)
        self.bn2r = nn.BatchNorm2d(inplanes)
        self.conv3r = nn.Conv2d(inplanes, inplanes, kernel_size=1,
bias=False)
        self.bn3r = nn.BatchNorm2d(inplanes)

        self.conv1l = nn.Conv2d(inplanes, inplanes, kernel_size=3,
```

```
stride=2, padding=1, bias=False, groups=inplanes)
        self.bn1l = nn.BatchNorm2d(inplanes)
        self.conv2l = nn.Conv2d(inplanes, inplanes, kernel_size=1,
bias=False)
        self.bn2l = nn.BatchNorm2d(inplanes)
        self.activation = activation(inplace=True)

        self.groups = groups
        self.inplanes = inplanes

    def forward(self, x):
        out_r = self.conv1r(x)
        out_r = self.bn1r(out_r)
        out_r = self.activation(out_r)

        out_r = self.conv2r(out_r)
        out_r = self.bn2r(out_r)

        out_r = self.conv3r(out_r)
        out_r = self.bn3r(out_r)
        out_r = self.activation(out_r)

        out_l = self.conv1l(x)
        out_l = self.bn1l(out_l)

        out_l = self.conv2l(out_l)
        out_l = self.bn2l(out_l)
        out_l = self.activation(out_l)

        return channel_shuffle(torch.cat((out_r, out_l), 1),
self.groups)


class ShuffleNetV2(nn.Module):
    """ShuffleNetV2 implementation.
    """

    def __init__(self, scale=1.0, in_channels=3, c_tag=0.5,
num_classes=1000, activation=nn.ReLU,
                 SE=False, residual=False, groups=2):
        """
        ShuffleNetV2 constructor
        :param scale:
        :param in_channels:
```

```python
        :param c_tag:
        :param num_classes:
        :param activation:
        :param SE:
        :param residual:
        :param groups:
        """

        super(ShuffleNetV2, self).__init__()

        self.scale = scale
        self.c_tag = c_tag
        self.residual = residual
        self.SE = SE
        self.groups = groups

        self.activation_type = activation
        self.activation = activation(inplace=True)
        self.num_classes = num_classes

        self.num_of_channels = {0.5: [24, 48, 96, 192, 1024], 1: [24,
116, 232, 464, 1024],
                                1.5: [24, 176, 352, 704, 1024], 2: [24,
244, 488, 976, 2048]}
        self.c = [_make_divisible(chan, groups) for chan in
self.num_of_channels[scale]]
        self.n = [3, 8, 3]  # TODO: should be [3,7,3]
        self.conv1 = nn.Conv2d(in_channels, self.c[0], kernel_size=3,
bias=False, stride=2, padding=1)
        self.bn1 = nn.BatchNorm2d(self.c[0])
        self.maxpool = nn.MaxPool2d(kernel_size=3, stride=2)
        self.shuffles = self._make_shuffles()

        self.conv_last = nn.Conv2d(self.c[-2], self.c[-1],
kernel_size=1, bias=False)
        self.bn_last = nn.BatchNorm2d(self.c[-1])
        self.avgpool = nn.AdaptiveAvgPool2d(1)
        self.fc = nn.Linear(self.c[-1], self.num_classes)
        self.init_params()

    def init_params(self):
        for m in self.modules():
            if isinstance(m, nn.Conv2d):
                init.kaiming_normal_(m.weight, mode='fan_out')
```

```
                if m.bias is not None:
                    init.constant_(m.bias, 0)
            elif isinstance(m, nn.BatchNorm2d):
                init.constant_(m.weight, 1)
                init.constant_(m.bias, 0)
            elif isinstance(m, nn.Linear):
                init.normal_(m.weight, std=0.001)
                if m.bias is not None:
                    init.constant_(m.bias, 0)

    def _make_stage(self, inplanes, outplanes, n, stage):
        modules = OrderedDict()
        stage_name = "ShuffleUnit{}".format(stage)

        # First module is the only one utilizing stride
        first_module = DownsampleUnit(inplanes=inplanes,
activation=self.activation_type, c_tag=self.c_tag,
                                      groups=self.groups)
        modules["DownsampleUnit"] = first_module
        second_module = BasicUnit(inplanes=inplanes * 2,
outplanes=outplanes, activation=self.activation_type,
                                  c_tag=self.c_tag, SE=self.SE,
residual=self.residual, groups=self.groups)
        modules[stage_name + "_{}".format(0)] = second_module
        # add more LinearBottleneck depending on number of repeats
        for i in range(n - 1):
            name = stage_name + "_{}".format(i + 1)
            module = BasicUnit(inplanes=outplanes, outplanes=outplanes,
activation=self.activation_type,
                               c_tag=self.c_tag, SE=self.SE,
residual=self.residual, groups=self.groups)
            modules[name] = module

        return nn.Sequential(modules)

    def _make_shuffles(self):
        modules = OrderedDict()
        stage_name = "ShuffleConvs"

        for i in range(len(self.c) - 2):
            name = stage_name + "_{}".format(i)
            module = self._make_stage(inplanes=self.c[i],
outplanes=self.c[i + 1], n=self.n[i], stage=i)
            modules[name] = module
```

```
        return nn.Sequential(modules)

    def forward(self, x):
        x = self.conv1(x)
        x = self.bn1(x)
        x = self.activation(x)
        x = self.maxpool(x)

        x = self.shuffles(x)
        x = self.conv_last(x)
        x = self.bn_last(x)
        x = self.activation(x)

        # average pooling layer
        x = self.avgpool(x)

        # flatten for input to fully-connected layer
        x = x.view(x.size(0), -1)
        x = self.fc(x)
        return F.log_softmax(x, dim=1)


if __name__ == "__main__":
    """Testing
    """
    model1 = ShuffleNetV2()
    print(model1)
    model2 = ShuffleNetV2(scale=0.5, in_channels=3, c_tag=0.5,
num_classes=1000, activation=nn.ReLU,
                          SE=False, residual=False)
    print(model2)
    model3 = ShuffleNetV2(in_channels=2, num_classes=10)
    print(model3)
    x = torch.randn(1, 2, 224, 224)
    print(model3(x))
    model4 = ShuffleNetV2( num_classes=10, groups=3, c_tag=0.2)
    print(model4)
    model4_size = 769
    x2 = torch.randn(1, 3, model4_size, model4_size, )
    print(model4(x2))
    model5 = ShuffleNetV2(scale=2.0,num_classes=10, SE=True,
residual=True)
```

```
x3 = torch.randn(1, 3, 196, 196)
print(model5(x3))
```
