# Supplementary material for: 4D printed deformation labels with machine learning for monitoring and preservation of respiring climacteric fruits
Source: Nat Commun. 2025 Nov 21;16:11525. doi: 10.1038/s41467-025-66554-6 (PMC12749378; doi:10.1038/s41467-025-66554-6)
Supplement: Supplementary file 4 — Supplementary Code [file 41467_2025_66554_MOESM4_ESM.zip › Supplementary Code/Code-train.pdf]

```
import os

import numpy as np
import torch
import torch.backends.cudnn as cudnn
import torch.distributed as dist
import torch.nn as nn
import torch.optim as optim
from torch.utils.data import DataLoader

from nets import get_model_from_name
from utils.callbacks import LossHistory
from utils.dataloader import DataGenerator, detection_collate
from utils.utils import (download_weights, get_classes,
get_lr_scheduler,
                         set_optimizer_lr, weights_init)
from utils.utils_fit import fit_one_epoch



if __name__ == "__main__":
    # ----------------------------------------------------#
    #   是否使用 Cuda
    #   没有 GPU 可以设置成 False
    # ----------------------------------------------------#
    Cuda = True  # 有 GPU
    #Cuda  = False
    # ---------------------------------------------------------------
------#
    #   distributed     用于指定是否使用单机多卡分布式运行
    #                   终端指令仅支持 Ubuntu。CUDA_VISIBLE_DEVICES 用于在
Ubuntu 下指定显卡。
    #                   Windows 系统下默认使用 DP 模式调用所有显卡，不支持 DDP。
    #   DP 模式:
    #       设置            distributed = False
    #       在终端中输入    CUDA_VISIBLE_DEVICES=0,1 python train.py
    #   DDP 模式:
    #       设置            distributed = True
    #       在终端中输入    CUDA_VISIBLE_DEVICES=0,1 python -m
torch.distributed.launch --nproc_per_node=2 train.py
    # ---------------------------------------------------------------
------#
    distributed = False
    # ---------------------------------------------------------------
```

```
------#
    #   sync_bn     是否使用 sync_bn，DDP 模式多卡可用
    # ------------------------------------------------------------------
------#
    sync_bn = False
    # ------------------------------------------------------------------
------#
    #   fp16        是否使用混合精度训练
    #               可减少约一半的显存、需要 pytorch1.7.1 以上
    # ------------------------------------------------------------------
------#
    fp16 = False
    # ----------------------------------------------------#
    #   训练自己的数据集的时候一定要注意修改 classes_path
    #   修改成自己对应的种类的 txt
    # ----------------------------------------------------#
    classes_path = 'folder_names.txt'
    # ----------------------------------------------------#
    #   输入的图片大小
    # ----------------------------------------------------#
    input_shape = [224, 224]
    # ------------------------------------------------------#
    #   所用模型种类：
    #   mobilenet、resnet50、vgg16、vit、已经其它类型的轻量级网络
    # ------------------------------------------------------#
    backbone        = "mobilenet"
    # backbone = "vit"
    # backbone = "mobilenetv1"
    #backbone = "ghostnet"
    # backbone = "cls_hrnet"
    #backbone = "shufflenet_v2"
    #backbone = "Xception"
    #backbone = "resnet50"
    #backbone = "vgg16"

    # ------------------------------------------------------------------
--------------------------------------------------------------#
    #   是否使用主干网络的预训练权重，此处使用的是主干的权重，因此是在模型构建的时
候进行加载的。
    #   如果设置了 model_path，则主干的权值无需加载，pretrained 的值无意义。
    #   如果不设置 model_path，pretrained = True，此时仅加载主干开始训练。
    #   如果不设置 model_path，pretrained = False，Freeze_Train = Fasle，
此时从 0 开始训练，且没有冻结主干的过程。
    # ------------------------------------------------------------------
```

```
--------------------------------------------------------------#
    pretrained      = True #有预训练模型
    #pretrained = False  # 无预训练模型
    # -----------------------------------------------------------------
--------------------------------------------------------------#

    # -----------------------------------------------------------------
--------------------------------------------------------------#
    # model_path      = "model_data/vit-patch_16.pth" #有预训练模型
    model_path = "model_data/mobilenet_v2-b0353104.pth" #有预训练模型
    model_path = ""  # 没有预训练模型
    # -----------------------------------------------------------------
--------------------------------------------------------------#

    # -----------------------------------------------------------------
---#
    #   冻结阶段训练参数
    #   此时模型的主干被冻结了，特征提取网络不发生改变
    #   占用的显存较小，仅对网络进行微调
    #   Init_Epoch          模型当前开始的训练世代，其值可以大于
Freeze_Epoch，如设置：
    #                       Init_Epoch = 60、Freeze_Epoch = 50、
UnFreeze_Epoch = 100
    #                       会跳过冻结阶段，直接从 60 代开始，并调整对应的学习率。
    #                       （断点续练时使用）
    #   Freeze_Epoch        模型冻结训练的 Freeze_Epoch
    #                       (当 Freeze_Train=False 时失效)
    #   Freeze_batch_size   模型冻结训练的 batch_size
    #                       (当 Freeze_Train=False 时失效)
    # -----------------------------------------------------------------
---#
    Init_Epoch = 0
    Freeze_Epoch = 50
    Freeze_batch_size = 4
    # -----------------------------------------------------------------
---#

    # -----------------------------------------------------------------
---#
    UnFreeze_Epoch = 800
    Unfreeze_batch_size = 4

    # -----------------------------------------------------------------
---#
```

```
    # Freeze_Train        = True #有预训练模型
    Freeze_Train = False
    # ------------------------------------------------------------------
---#
    #   其它训练参数：学习率、优化器、学习率下降有关
    # ------------------------------------------------------------------
---##
    Init_lr = 1e-2
    Min_lr = Init_lr * 0.01
    # ------------------------------------------------------------------
---#

    optimizer_type = "sgd"
    momentum = 0.9
    weight_decay = 5e-4
    # ------------------------------------------------------------------
---#
    #   lr_decay_type   使用到的学习率下降方式，可选的有 step、cos
    # ------------------------------------------------------------------
---#
    lr_decay_type = "step"
    # ------------------------------------------------------------------
---#
    #   save_period     多少个 epoch 保存一次权值，默认每个世代都保存
    # ------------------------------------------------------------------
---#
    save_period = 1
    # ------------------------------------------------------------------
---#
    #   save_dir        权值与日志文件保存的文件夹
    # ------------------------------------------------------------------
---#
    save_dir = 'logs'
    # ------------------------------------------------------------------
---#
    #   num_workers     用于设置是否使用多线程读取数据
    #                   开启后会加快数据读取速度，但是会占用更多内存
    #                   内存较小的电脑可以设置为 2 或者 0
    # ------------------------------------------------------------------
---#
    num_workers = 2

    # ---------------------------------------------------------#
    #   train_annotation_path   训练图片路径和标签
```

```
    #   test_annotation_path    验证图片路径和标签（使用测试集代替验证集）
    # ------------------------------------------------------#
    train_annotation_path = "cls_train.txt"
    test_annotation_path = 'cls_test.txt'

    # ------------------------------------------------------#
    #   设置用到的显卡
    # ------------------------------------------------------#
    ngpus_per_node = torch.cuda.device_count()
    if distributed:
        dist.init_process_group(backend="nccl")
        local_rank = int(os.environ["LOCAL_RANK"])
        rank = int(os.environ["RANK"])
        device = torch.device("cuda", local_rank)
        if local_rank == 0:
            print(f"[{os.getpid()}] (rank = {rank}, local_rank =
{local_rank}) training...")
            print("Gpu Device Count : ", ngpus_per_node)
    else:
        device = torch.device('cuda' if torch.cuda.is_available() else
'cpu')
        local_rank = 0
        rank = 0

    if pretrained:
        if distributed:
            if local_rank == 0:
                download_weights(backbone)
            dist.barrier()
        else:
            download_weights(backbone)

    # ------------------------------------------------------#
    #   获取 classes
    # ------------------------------------------------------#
    class_names, num_classes = get_classes(classes_path)

    # if backbone != "vit":
    #     model = get_model_from_name[backbone](num_classes =
num_classes, pretrained = pretrained)
    # else:
    #     model = get_model_from_name[backbone](input_shape =
input_shape, num_classes = num_classes, pretrained = pretrained)
```

```
    if backbone == "vit":
        model = get_model_from_name[backbone](input_shape=input_shape,
num_classes=num_classes, pretrained=pretrained)
    elif backbone == "mobilenet" or backbone == "mobilenetv1" or
backbone == "resnet50" or backbone == "vgg16":
        model = get_model_from_name[backbone](num_classes=num_classes,
pretrained=pretrained)
    # elif backbone == "ghostnet":
    #     model =
get_model_from_name[backbone](num_classes=num_classes)
    else:
        model = get_model_from_name[backbone](num_classes=num_classes)
    if not pretrained:
        weights_init(model)
    if model_path != "":
        if local_rank == 0:
            # ------------------------------------------------------#
            #   载入预训练权重
            # ------------------------------------------------------#
            print('Loading weights into state dict...')
        model_dict = model.state_dict()
        pretrained_dict = torch.load(model_path, map_location=device)
        pretrained_dict = {k: v for k, v in pretrained_dict.items() if
np.shape(model_dict[k]) == np.shape(v)}
        model_dict.update(pretrained_dict)
        model.load_state_dict(model_dict)

    if local_rank == 0:
        loss_history = LossHistory(save_dir, model,
input_shape=input_shape)
    else:
        loss_history = None

    if fp16:
        # ------------------------------------------------------------
------#
        #   torch 1.2 不支持 amp，建议使用 torch 1.7.1 及以上正确使用 fp16
        #   因此 torch1.2 这里显示"could not be resolve"
        # ------------------------------------------------------------
------#
        from torch.cuda.amp import GradScaler as GradScaler

        scaler = GradScaler()
    else:
```

```
        scaler = None

    model_train = model.train()
    # ----------------------------#
    #   多卡同步 Bn
    # ----------------------------#
    if sync_bn and ngpus_per_node > 1 and distributed:
        model_train =
torch.nn.SyncBatchNorm.convert_sync_batchnorm(model_train)
    elif sync_bn:
        print("Sync_bn is not support in one gpu or not distributed.")

    if Cuda:
        if distributed:
            # ----------------------------#
            #   多卡平行运行
            # ----------------------------#
            model_train = model_train.cuda(local_rank)
            model_train =
torch.nn.parallel.DistributedDataParallel(model_train,
device_ids=[local_rank],

find_unused_parameters=True)
        else:
            model_train = torch.nn.DataParallel(model)
            cudnn.benchmark = True
            model_train = model_train.cuda()

    # ---------------------------#
    #   读取数据集对应的 txt
    # ---------------------------#
    with open(train_annotation_path, encoding='MacRoman') as f:
        train_lines = f.readlines()
    with open(test_annotation_path, encoding='ascii') as f:
        val_lines = f.readlines()
    num_train = len(train_lines)
    num_val = len(val_lines)
    np.random.seed(10101)
    np.random.shuffle(train_lines)
    np.random.seed(None)



    # -------------------------------------------------------#
    if True:
```

```
        UnFreeze_flag = False
        # ------------------------------------#
        #   冻结一定部分训练
        # ------------------------------------#
        if Freeze_Train:
            model.freeze_backbone()

        # ------------------------------------------------------------------
-------#
        #   如果不冻结训练的话，直接设置 batch_size 为 Unfreeze_batch_size
        # ------------------------------------------------------------------
-------#
        batch_size = Freeze_batch_size if Freeze_Train else
Unfreeze_batch_size

        # ------------------------------------------------------------------
-------#
        #   判断当前 batch_size，自适应调整学习率
        # ------------------------------------------------------------------
-------#
        nbs = 64
        lr_limit_max = 1e-3 if optimizer_type == 'adam' else 1e-1
        lr_limit_min = 1e-4 if optimizer_type == 'adam' else 5e-4
        if backbone == 'vit':
            nbs = 256
            lr_limit_max = 1e-3 if optimizer_type == 'adam' else 1e-1
            lr_limit_min = 1e-5 if optimizer_type == 'adam' else 5e-4
        Init_lr_fit = min(max(batch_size / nbs * Init_lr,
lr_limit_min), lr_limit_max)
        Min_lr_fit = min(max(batch_size / nbs * Min_lr, lr_limit_min *
1e-2), lr_limit_max * 1e-2)

        optimizer = {
            'adam': optim.Adam(model_train.parameters(), Init_lr_fit,
betas=(momentum, 0.999),
                               weight_decay=weight_decay),
            'sgd': optim.SGD(model_train.parameters(), Init_lr_fit,
momentum=momentum, nesterov=True)
        }[optimizer_type]

        # ------------------------------------------#
        #   获得学习率下降的公式
        # ------------------------------------------#
        lr_scheduler_func = get_lr_scheduler(lr_decay_type,
```

```
Init_lr_fit, Min_lr_fit, UnFreeze_Epoch)

        # ---------------------------------------#
        #   判断每一个世代的长度
        # ---------------------------------------#
        epoch_step = num_train // batch_size
        epoch_step_val = num_val // batch_size

        if epoch_step == 0 or epoch_step_val == 0:
            raise ValueError("数据集过小，无法继续进行训练，请扩充数据集。")

        train_dataset = DataGenerator(train_lines, input_shape, True)
        val_dataset = DataGenerator(val_lines, input_shape, False)

        if distributed:
            train_sampler =
torch.utils.data.distributed.DistributedSampler(train_dataset,
shuffle=True, )
            val_sampler =
torch.utils.data.distributed.DistributedSampler(val_dataset,
shuffle=False, )
            batch_size = batch_size // ngpus_per_node
            shuffle = False
        else:
            train_sampler = None
            val_sampler = None
            shuffle = True

        gen = DataLoader(train_dataset, shuffle=shuffle,
batch_size=batch_size, num_workers=num_workers,
                         pin_memory=True,
                         drop_last=True, collate_fn=detection_collate,
sampler=train_sampler)
        gen_val = DataLoader(val_dataset, shuffle=shuffle,
batch_size=batch_size, num_workers=num_workers,
                             pin_memory=True,
                             drop_last=True, collate_fn=detection_collate,
sampler=val_sampler)
        log_file = os.path.join(save_dir, 'training_log.txt')
        with open(log_file, 'w') as log:
            log.write("epoch, train_loss, train_accuracy\n")
        # ---------------------------------------#
        #   开始模型训练
        # ---------------------------------------#
```

```
        for epoch in range(Init_Epoch, UnFreeze_Epoch):
            # ---------------------------------------#
            #   如果模型有冻结学习部分
            #   则解冻，并设置参数
            # ---------------------------------------#
            if epoch >= Freeze_Epoch and not UnFreeze_flag and
Freeze_Train:
                batch_size = Unfreeze_batch_size

                # -------------------------------------------------------
---------------#
                #   判断当前 batch_size，自适应调整学习率
                # -------------------------------------------------------
---------------#
                nbs = 64
                lr_limit_max = 1e-3 if optimizer_type == 'adam' else
1e-1
                lr_limit_min = 1e-4 if optimizer_type == 'adam' else
5e-4
                if backbone == 'vit':
                    nbs = 256
                    lr_limit_max = 1e-3 if optimizer_type == 'adam' else
1e-1
                    lr_limit_min = 1e-5 if optimizer_type == 'adam' else
5e-4
                Init_lr_fit = min(max(batch_size / nbs * Init_lr,
lr_limit_min), lr_limit_max)
                Min_lr_fit = min(max(batch_size / nbs * Min_lr,
lr_limit_min * 1e-2), lr_limit_max * 1e-2)
                # ---------------------------------------#
                #   获得学习率下降的公式
                # ---------------------------------------#
                lr_scheduler_func = get_lr_scheduler(lr_decay_type,
Init_lr_fit, Min_lr_fit, UnFreeze_Epoch)

                model.Unfreeze_backbone()

                epoch_step = num_train // batch_size
                epoch_step_val = num_val // batch_size

                if epoch_step == 0 or epoch_step_val == 0:
                    raise ValueError("数据集过小，无法继续进行训练，请扩充数据
集。")
```

```
            if distributed:
                batch_size = batch_size // ngpus_per_node

            gen = DataLoader(train_dataset, shuffle=shuffle,
batch_size=batch_size, num_workers=num_workers,
                             pin_memory=True,
                             drop_last=True,
collate_fn=detection_collate, sampler=train_sampler)
            gen_val = DataLoader(val_dataset, shuffle=shuffle,
batch_size=batch_size, num_workers=num_workers,
                                 pin_memory=True,
                                 drop_last=True,
collate_fn=detection_collate, sampler=val_sampler)

            UnFreeze_flag = True

        if distributed:
            train_sampler.set_epoch(epoch)

        set_optimizer_lr(optimizer, lr_scheduler_func, epoch)

        '''fit_one_epoch(model_train, model, loss_history,
optimizer, epoch, epoch_step, epoch_step_val, gen, gen_val,
                      UnFreeze_Epoch, Cuda, fp16, scaler,
save_period, save_dir, local_rank)'''

        train_loss, train_accuracy = fit_one_epoch(model_train,
model, loss_history, optimizer, epoch, epoch_step,
                                                epoch_step_val, gen,
gen_val, UnFreeze_Epoch, Cuda, fp16, scaler,
                                                save_period, save_dir,
local_rank)

        with open(log_file, 'a') as log:
            log.write(f"{epoch}, {train_loss:.4f},
{train_accuracy:.4f}\n")

    if local_rank == 0:
        loss_history.writer.close()
```
