# Supplementary material for: 4D printed deformation labels with machine learning for monitoring and preservation of respiring climacteric fruits
Source: Nat Commun. 2025 Nov 21;16:11525. doi: 10.1038/s41467-025-66554-6 (PMC12749378; doi:10.1038/s41467-025-66554-6)
Supplement: Supplementary file 4 — Supplementary Code [file 41467_2025_66554_MOESM4_ESM.zip › Supplementary Code/Code-Xception.pdf]

```
import torch
import torch.nn as nn
import torch.nn.functional as F

class SeparableConv2d(nn.Module):
    def __init__(self, in_channels, out_channels, kernel_size=1,
stride=1, padding=0, dilation=1):
        super(SeparableConv2d, self).__init__()

        self.conv1 = nn.Conv2d(
            in_channels, in_channels, kernel_size, stride, padding,
dilation, groups=in_channels, bias=False)
        self.pointwise = nn.Conv2d(in_channels, out_channels, 1, 1, 0,
1, 1, bias=False)

    def forward(self, x):
        x = self.conv1(x)
        x = self.pointwise(x)
        return x


class Block(nn.Module):
    def __init__(self, in_channels, out_channels, reps, strides=1,
start_with_relu=True, grow_first=True):
        super(Block, self).__init__()

        if out_channels != in_channels or strides != 1:
            self.skip = nn.Conv2d(in_channels, out_channels, 1,
stride=strides, bias=False)
            self.skipbn = nn.BatchNorm2d(out_channels)
        else:
            self.skip = None

        rep = []
        for i in range(reps):
            if grow_first:
                inc = in_channels if i == 0 else out_channels
                outc = out_channels
            else:
                inc = in_channels
                outc = in_channels if i < (reps - 1) else out_channels
            rep.append(nn.ReLU(inplace=True))
            rep.append(SeparableConv2d(inc, outc, 3, stride=1,
padding=1))
```

```python
            rep.append(nn.BatchNorm2d(outc))

        if not start_with_relu:
            rep = rep[1:]
        else:
            rep[0] = nn.ReLU(inplace=False)

        if strides != 1:
            rep.append(nn.MaxPool2d(3, strides, 1))
        self.rep = nn.Sequential(*rep)

    def forward(self, inp):
        x = self.rep(inp)

        if self.skip is not None:
            skip = self.skip(inp)
            skip = self.skipbn(skip)
        else:
            skip = inp

        x += skip
        return x


class Xception(nn.Module):
    """
    Xception optimized for the ImageNet dataset, as specified in
    https://arxiv.org/pdf/1610.02357.pdf
    """

    def __init__(self, num_classes=1000, in_chans=3, drop_rate=0.,
global_pool='avg'):
        """ Constructor
        Args:
            num_classes: number of classes
        """
        super(Xception, self).__init__()
        self.drop_rate = drop_rate
        self.global_pool = global_pool
        self.num_classes = num_classes
        self.num_features = 2048

        self.conv1 = nn.Conv2d(in_chans, 32, 3, 2, 0, bias=False)
        self.bn1 = nn.BatchNorm2d(32)
```

```
        self.act1 = nn.ReLU(inplace=True)

        self.conv2 = nn.Conv2d(32, 64, 3, bias=False)
        self.bn2 = nn.BatchNorm2d(64)
        self.act2 = nn.ReLU(inplace=True)

        self.block1 = Block(64, 128, 2, 2, start_with_relu=False)
        self.block2 = Block(128, 256, 2, 2)
        self.block3 = Block(256, 728, 2, 2)

        self.block4 = Block(728, 728, 3, 1)
        self.block5 = Block(728, 728, 3, 1)
        self.block6 = Block(728, 728, 3, 1)
        self.block7 = Block(728, 728, 3, 1)

        self.block8 = Block(728, 728, 3, 1)
        self.block9 = Block(728, 728, 3, 1)
        self.block10 = Block(728, 728, 3, 1)
        self.block11 = Block(728, 728, 3, 1)

        self.block12 = Block(728, 1024, 2, 2, grow_first=False)

        self.conv3 = SeparableConv2d(1024, 1536, 3, 1, 1)
        self.bn3 = nn.BatchNorm2d(1536)
        self.act3 = nn.ReLU(inplace=True)

        self.conv4 = SeparableConv2d(1536, self.num_features, 3, 1, 1)
        self.bn4 = nn.BatchNorm2d(self.num_features)
        self.act4 = nn.ReLU(inplace=True)
        self.feature_info = [
            dict(num_chs=64, reduction=2, module='act2'),
            dict(num_chs=128, reduction=4, module='block2.rep.0'),
            dict(num_chs=256, reduction=8, module='block3.rep.0'),
            dict(num_chs=728, reduction=16, module='block12.rep.0'),
            dict(num_chs=2048, reduction=32, module='act4'),
        ]

        self.fc = nn.Linear(self.num_features, self.num_classes)

        # #------- init weights --------
        for m in self.modules():
            if isinstance(m, nn.Conv2d):
                nn.init.kaiming_normal_(m.weight, mode='fan_out',
nonlinearity='relu')
```

```python
        elif isinstance(m, nn.BatchNorm2d):
            m.weight.data.fill_(1)
            m.bias.data.zero_()

    def forward_features(self, x):
        x = self.conv1(x)
        x = self.bn1(x)
        x = self.act1(x)

        x = self.conv2(x)
        x = self.bn2(x)
        x = self.act2(x)

        x = self.block1(x)
        x = self.block2(x)
        x = self.block3(x)
        x = self.block4(x)
        x = self.block5(x)
        x = self.block6(x)
        x = self.block7(x)
        x = self.block8(x)
        x = self.block9(x)
        x = self.block10(x)
        x = self.block11(x)
        x = self.block12(x)

        x = self.conv3(x)
        x = self.bn3(x)
        x = self.act3(x)

        x = self.conv4(x)
        x = self.bn4(x)
        x = self.act4(x)
        return x

    def forward(self, x):
        x = self.forward_features(x)
        adaptiveAvgPoolWidth = x.shape[2]
        x = F.avg_pool2d(x, kernel_size=adaptiveAvgPoolWidth)
        if self.drop_rate > 0:
            x = F.dropout(x, p=self.drop_rate, training=self.training)
        x = x.view(x.size(0), -1)
        x = self.fc(x)
        return x
```

```
def xception():
    return Xception()
```
