# Supplementary material for: 4D printed deformation labels with machine learning for monitoring and preservation of respiring climacteric fruits
Source: Nat Commun. 2025 Nov 21;16:11525. doi: 10.1038/s41467-025-66554-6 (PMC12749378; doi:10.1038/s41467-025-66554-6)
Supplement: Supplementary file 5 — Reporting Summary [file 41467_2025_66554_MOESM5_ESM.pdf]

nature portfolio

Corresponding author(s): DAPR-NCOMMS-25-20212B

Last updated by author(s): Oct 17, 2025

Reporting Summary

Nature Portfolio wishes to improve the reproducibility of the work that we publish. This form provides structure for consistency and transparency in reporting. For further information on Nature Portfolio policies, see our Editorial Policies and the Editorial Policy Checklist.

Statistics

For all statistical analyses, confirm that the following items are present in the figure legend, table legend, main text, or Methods section.

n/a	Confirmed	
☐	☒	The exact sample size (*n*) for each experimental group/condition, given as a discrete number and unit of measurement
☐	☒	A statement on whether measurements were taken from distinct samples or whether the same sample was measured repeatedly
☐	☒	The statistical test(s) used AND whether they are one- or two-sided<br>*Only common tests should be described solely by name; describe more complex techniques in the Methods section.*
☐	☒	A description of all covariates tested
☐	☒	A description of any assumptions or corrections, such as tests of normality and adjustment for multiple comparisons
☐	☒	A full description of the statistical parameters including central tendency (e.g. means) or other basic estimates (e.g. regression coefficient) AND variation (e.g. standard deviation) or associated estimates of uncertainty (e.g. confidence intervals)
☐	☒	For null hypothesis testing, the test statistic (e.g. *F*, *t*, *r*) with confidence intervals, effect sizes, degrees of freedom and *P* value noted<br>*Give P values as exact values whenever suitable.*
☒	☐	For Bayesian analysis, information on the choice of priors and Markov chain Monte Carlo settings
☐	☒	For hierarchical and complex designs, identification of the appropriate level for tests and full reporting of outcomes
☐	☒	Estimates of effect sizes (e.g. Cohen's *d*, Pearson's *r*), indicating how they were calculated

Our web collection on statistics for biologists contains articles on many of the points above.

Software and code

Policy information about availability of computer code

Data collection	Origin 2022 software (OriginLab Corporation, Massachusetts, USA)
Data analysis	Rhinoceros 5.0 (Robert McNeel & Associates, Washington, USA)， Repetier-Host software (Hot-World GmbH & Co. KG, Würzburg, Germany)， Image-Pro Plus 6.0 software (Media Cybernetics, Silver Spring, USA)， PyCharm Community Edition 2023 (JetBrains, Prague, Czech Republic)， Origin 2022 software (OriginLab Corporation, Massachusetts, USA) and IBM SPSS Statistics 28 software (IBM Corporation, New York, USA).

For manuscripts utilizing custom algorithms or software that are central to the research but not yet described in published literature, software must be made available to editors and reviewers. We strongly encourage code deposition in a community repository (e.g. GitHub). See the Nature Portfolio guidelines for submitting code & software for further information.

Data

Policy information about availability of data

All manuscripts must include a data availability statement. This statement should provide the following information, where applicable:

- Accession codes, unique identifiers, or web links for publicly available datasets
- A description of any restrictions on data availability
- For clinical datasets or third party data, please ensure that the statement adheres to our policy

This study doesn’t involve clinical or third-party data. There are no restrictions on the availability of the data. All data are freely available for public use.

Research involving human participants, their data, or biological material

Policy information about studies with human participants or human data. See also policy information about sex, gender (identity/presentation), and sexual orientation and race, ethnicity and racism.

Reporting on sex and gender	The experimental part of this study involving human participants was the sensory evaluation of fruit quality. The participants were 10 trained volunteers from our research institution. The ten volunteers included four males and six females based on self-assessment according to biological attributes. Since this study did not involve research in areas such as medicine, clinical studies, psychology, and genetics, etc., sex and gender identity analysis was not conducted. When selecting volunteers, the primary criteria were the reliability of the test results and the mastery of the evaluation standards.
Reporting on race, ethnicity, or other socially relevant groupings	The experimental part of this study did not involve the analysis of race, ethnicity, or other socially relevant groupings, because this study did not include research in areas such as medicine, clinical studies, psychology, and genetics, etc.
Population characteristics	See above.
Recruitment	The sensory evaluation volunteers were recruited from our research institution. They underwent sensory training, and 10 individuals were selected based on the reliability of the results and the mastery of the evaluation standards. To ensure diversity, the volunteers included 4 males and 6 females. Considering sensory sensitivity, the age of the volunteers ranged from 20 to 38 years old. The results of this sensory evaluation were reliable.
Ethics oversight	This study involves the sensory test of the quality of fruits. Sensory evaluation is conducted at the Research Center of Food Resources and Comprehensive Utilization of Jiangnan University. At present, the ethical examination and approval of sensory evaluation experiments is not stipulated and required by Chinese laws. The School of Food Science and Technology of Jiangnan University can prove that the research meets the local moral and ethical standards and does not require ethical approval.

Note that full information on the approval of the study protocol must also be provided in the manuscript.

Field-specific reporting

Please select the one below that is the best fit for your research. If you are not sure, read the appropriate sections before making your selection.

☐ Life sciences ☐ Behavioural & social sciences ☒ Ecological, evolutionary & environmental sciences

For a reference copy of the document with all sections, see nature.com/documents/nr-reporting-summary-flat.pdf

Ecological, evolutionary & environmental sciences study design

All studies must disclose on these points even when the disclosure is negative.

Study description	This manuscript presented the first study on 4D printed pH-responsive smart labels for monitoring fruit freshness and their preservation effect. The effects of different formulations and different printing structure on deformation of labels were evaluated. The positive significance of deformation in freshness monitoring and preservation was elucidated. In terms of their monitoring and preservation effects, the differences between traditional labels, 3D printed labels, and 4D printed labels were compared, and the underlying mechanisms were analyzed. The recognition accuracy of four lightweight deep convolutional neural networks models for labels was tested to select the optimal label-model combination. The recognition accuracy of this optimal combination for fruit freshness was also evaluated.
Research sample	The objects in this study included the printed labels with different essential oil contents or structural designs. The selection of materials was primarily based on commonly available materials in the market. The test fruits were Kiwi fruit (Actinidia chinensis Planch.), green mango (Mangifera indica Linn.) and persimmon (Diospyros kaki Thunb.), all of which were climacteric fruits. These fruits were harvested from plantation at Yantai (Shandong, China), Changjiang (Hainan, China), and Baoding (Hebei, China), respectively. When selecting the fruits, those with uniform size, consistent ripeness, and no mechanical damage were chosen.
Sampling strategy	Each test indicator was assessed using three samples, and each test indicator was repeated three times. If the results were consistent and showed high repeatability, the data were recorded. If deviations occurred, we conducted an additional seven tests, with each test including three samples. Data with a repeatability rate of 95% or higher were selected.
Data collection	The first author of this manuscript recorded the data using Origin 2022 software (OriginLab Corporation, Massachusetts, USA).
Timing and spatial scale	In this manuscript, the sampling intervals and periodicity varied according to the different measurement indicators. The specific sampling intervals and periodicity were detailed in the Methods section and illustrated in the figures and tables of this manuscript.
Data exclusions	When testing a specific physicochemical property of the labels, occasional measurements were significantly deviate from the previously measured data set during repetitions. In such cases, the indicator was retested seven times, with each test including three samples. Based on the distribution of the data set and the additional test data, the decision to exclude the severely deviated data was made. The primary cause of the severe deviation was due to testing operation errors.

Reproducibility	Each indicator was measured at least three times, with each measurement including three samples. During the measurement process, occasional data deviated from the data set due to human error. In such cases, an additional seven repeated tests were conducted. The inclusion or exclusion of these outlier data points was determined based on the data distribution. In the study, all repeatability tests for the measurements of the indicators were successful.
Randomization	When testing the practical application of the labels, the fruits were selected from the plantation and needed to be uniform in ripeness, similar in shape, and free of mechanical damage. The selected fruits were randomly allocated into the packaging boxes according to the total weight requirements.
Blinding	In the research process, information was concealed from the personnel involved in the measurement of indicators, data collection, processing, and analysis.

Did the study involve field work? ☐ Yes ☒ No

Reporting for specific materials, systems and methods

We require information from authors about some types of materials, experimental systems and methods used in many studies. Here, indicate whether each material, system or method listed is relevant to your study. If you are not sure if a list item applies to your research, read the appropriate section before selecting a response.

Materials & experimental systems

n/a	Involved in the study
☒	☐ Antibodies
☒	☐ Eukaryotic cell lines
☒	☐ Palaeontology and archaeology
☒	☐ Animals and other organisms
☒	☐ Clinical data
☒	☐ Dual use research of concern
☒	☐ Plants

Methods

n/a	Involved in the study
☒	☐ ChIP-seq
☒	☐ Flow cytometry
☒	☐ MRI-based neuroimaging

Plants

Seed stocks	The test fruits were Kiwi fruit (Actinidia chinensis Planch.), green mango (Mangifera indica Linn.) and persimmon (Diospyros kaki Thunb.), all of which were climacteric fruits. These fruits were harvested from plantation at Yantai (Shandonirg, China), Changjiang (Hainan, China), and Baoding (Hebei, China), respectively. Their harvesting dates were on 5 September, 21 May, and 12 September, 2023. When selecting the fruits, those with uniform size, consistent ripeness, and no mechanical damage were chosen.
Novel plant genotypes	This study was not concerned with generating novel plant genotypes.
Authentication	This study did not involve the use of seed stocks or the generation of novel genotypes.
